# Efficient extraction of oleoresin from *Ferula gummosa* roots by natural deep eutectic solvent and its structure and chemical characterizations

**DOI:** 10.1038/s41598-023-46198-6

**Published:** 2024-01-02

**Authors:** Javad Radmard, Ali Mohamadi Sani, Akram Arianfar, Behrooz Mahmoodzadeh Vaziri

**Affiliations:** 1 Department of Food Science and Technology, Quchan Branch, Islamic Azad University, Quchan, Iran; 2 Department of Chemical Engineering, Quchan Branch, Islamic Azad University, Quchan, Iran

**Keywords:** Environmental sciences, Chemistry

## Abstract

Deep eutectic solvents in the extraction of plant metabolites have found many advantages, such as low toxicity, biodegradability, low cost and ease of preparation over the conventional methods. This work aims to compare natural deep eutectic solvents in extraction and optimization of oleoresin from *Ferula gummosa* and determining its chemical and structure properties. Box–Behnken design was applied to optimize the extraction of oleoresin from *Ferula gummosa* using eutectic solvents. The variables of extraction were extraction time, temperature, and ratio of eutectic solvents. Six mixtures of eutectic solvents including choline chloride/urea, acetic acid, lactic acid, formic acid, formamide and glycerol at ratios of 2:1 and 3:1 were evaluated. The highest yields were obtained for choline chloride/formic acid, choline chloride/formamide. The quadratic regression equation was set up as a predictive model with an R^2^ value of 0.85. The optimum condition was 6 h, 40 °C, and ratio 12.5% (w/v). No significant difference was found between the predicted and experimental yield. The main components of the oleoresin were β-pinene (40.27%), cylcofenchen (11.93%) and α-pinene (7.53%) as characterized by gas chromatography-mass spectrometry. The chemical structure study by spectroscopy showed that no solvents remained in the oleoresin. Therefore, *F. gummosa* oleoresin can be explored as a novel promising natural pharmaceutical ingredient extracted with eutectic solvents.

## Introduction

*Ferula gummosa* Boiss. is a native indigenous plant that grows mainly in the north and southwest of Iran and is known as “Barijeh”. Ferula essential oil is an effective ingredient in treatment of diarrhea and has spasmolytic activity^1^. Due to its antispasmodic, expectorant, anticonvulsant, anti-catarrhal, anti-nociceptive, and antimicrobial properties, it is also used in the adhesive, paint, cosmetic, perfume, and pharmaceutical industries^2,3^. It has also been used as flavoring agent or emulsifier in foods and beverages^4,5^. The chemical compositions of all essential oils of *F. gummosa* have shown that it contains mainly components of α- and β-pinene^6–9^. Several studies of the volatile components of *F. gummosa* oleoresin have shown that the major fraction consists of monoterpene hydrocarbons (~ 73–88%)^2,10^.

Environmentally friendly extraction technology from natural products is essential and is a high priority in chemistry due to the environmental sustainability and reduced negative impact on humans^11,12^. Natural deep eutectic solvents (NADES) are green solvents that can be used in many chemical processes, such as extraction^13,14^. NADES has many advantages over conventional solvents, such as safety, economy, inflammability, biodegradability, thermal and chemical stability, environmental friendliness, and food grade, and no further procedures are required to isolate and purify the compound^15,16^. DES is a mixture of two or more solvents that, in a given composition, exhibit a strong reduction in melting point and become liquid at ambient temperature^17^. NADES components are primary metabolites such as sugars (glucose, sucrose, and fructose), organic acids (lactic, malic, and citric acids), urea, and quaternary ammonium salts (choline chloride or betaine)^18–21^. NADES has been applied in the extraction of widely used metabolites, polar and nonpolar compounds, from plants such as green coffee beans^12,22,23^, *Pseudowintera colorata*^24^, *Ginkgo biloba*^25^, mangosteen pericarp^26^, cannabis^27^, *Olea europaea*^28^, *Citrus grandis* L. Osbeck^29^, *Herba Artemisiae Scopariae*^30^*, Cynara cardunculus* L.^31^, *Carthamus tinctorius*^32^*, Tartary buckwheat hull*^33^*,* fig leaves^34^, *Sophora japonica*^35^, and *Cajanus cajan*^36^*.* Furthermore, the deacidification of palm and soybean oil to preserve antioxidants have been accomplished by NADES^37,38^.

The selection of the right solvents is critical to the success of the extraction process^23^. Choline chloride as a hydrogen bond acceptor (HBA) has received considerable attention due to its innocuousness, biocompatibility, and biodegradability. Furthermore, various hydrogen bond donors (HBD) such as urea, acetic acid, lactic acid, formic acid, and glycerol have also been used together with HBD to develop a eutectic solvent^39^. In this regard, the mixture of choline chloride with other solvents has been shown to improve the extraction yield compared to other conventional extractions^18,36^. Moreover, the mixture of choline chloride-lactic acid has shown higher extraction efficiency in the extraction of flavonoids from *Radix Scutellariae*^40^ and choline chloride-alcohol for the extraction of phenolic acids^41^.

Despite numerous research works on DES extraction of different bioactive compounds such as anthocyanins, polyphenols, flavonoids, and catechins^42^, to the best of our knowledge, no research work has been conducted on the extraction of oleoresin from *F. gummosa* using eutectic solvents. Therefore, the aim of the present work is to apply tailored DES to extract the oleoresin from *F. gummosa,* compare it with the conventional method, and characterize the chemical and structure of *F. gummosa* oleoresin by GC and FTIR spectroscopy.

## Materials and methods

### Materials

*Ferula gummosa* was collected in September 2021 at an elevation of approximately 2340 m above sea level in Farouj, Iran. A voucher specimen was sent to the herbarium of the Research Institute of Forests and Rangelands (TARI), Tehran, Iran. According to the previous work of Jalali^2,6,9,10^, the soil around the lower part of the herb stem was removed and the surface near the root was scratched to collect the exudate in a stainless steel container for one week. The oleoresin was obtained from approximately 100 healthy, 4–6 year old plants. The exudates were mixed and stored in a hermetic plastic container in a refrigerator (4 °C). The eutectic solvents, including analytical grade choline chloride (CL) (99% purity) as HBA and urea (purity 99–100%), lactic acid (purity > 85%), acetic acid (purity > 85%), formic acid (purity > 85%), and glycerol (purity > 90%), as HBDs, were obtained from Merck (Darmstadt, Germany).

### DES synthesis

DES were prepared following previous work with some modifications^43^. The solutions of CL-urea, CL-acetic acid, CL-lactic acid, and CL-glycerol were prepared in a 2:1 ratio, while CL-formic acid and CL-formamide were obtained in a 3:1 ratio. The molar ratio of DES was chosen based on our preliminary experiments. Choline chloride (HBA) and other solvents such as HBD were mixed separately in an optimized molar ratio in a reactor and heated at 90 °C for 4 h via a magnet stirrer at 400 rpm until a transparent liquid was obtained. The DES was stored in a glass with a screw cap vial in the dark condition. The DES samples were examined intermittently over a period of several weeks for the appearance of the crystals.

### Extraction of *F. gummosa* oleoresin

#### Conventional extraction

The oleoresin was extracted from the exudates by hydrodistillation in a Clevenger-type apparatus for 3 h and recovered with diethyl ether, dried over anhydrous sodium sulphate, and the solvent carefully removed with a rotary evaporator. The oleoresin was stored at 4 °C until further analysis.

#### Extraction of *F. gummosa* oleoresin using DES

All 6 DES mixtures were used separately to extract *F. gummosa* oleoresin*.* In a typical experiment, 1000 mg of *F. gummosa* exudates were taken separately in 5 g DES in a beaker. The mixture was then heated at 30–50 °C for various periods of time (3, 6, and 9 h). The mixtures were centrifuged at 3000 rpm for 5 min and the mass obtained at the bottom of the centrifuge tube was separated, washed several times with IPA and dried under vacuum. In a control experiment, 500 mg of *F. gummosa* exudates were mixed with 10 ml of ethanol, keeping the temperature and time the same as in the previous reactions. The supernatant was precipitated in IPA (1:3 v/v). The precipitated oleoresin dried in vacuum.

### Experimental design

The effects of three factors, time (3–9 h), temperature (30–50 °C), and DES percentage (5–20% w/v), on the extraction of *F. gummosa* oleoresin were analyzed using a single-factor experimental design performed with Design-Expert version 13 (Statease Inc., Minneapolis, MN, USA). In each experiment, a single factor was changed while the other factors remained constant. A three-level Box–Behnken design (BBD) was further optimized. Time (A), temperature (B), and DES content (C) were independent variables, while oleoresin extraction yield was the dependent variable. The values of the variables are given in Table 1.

**Table 1:** Coded and actual levels of independent variables used in BBD design.

Independent variables	Symbol	Unit	levels		
			− 1	0	1
Time	t	Hour	3	6	9
Temperature	T	°C	30	40	50
DES solvent	S	%	5	10	20

### GC–MS analysis

Gas chromatography–mass spectrometry analysis (GC–MS) was performed using a gas chromatograph (7890B, Agilent, Santa Clara, CA, USA) connected to a mass detector (5977A, Agilent technologies, USA). The gas chromatograph was equipped with a HP 5-ms capillary column (phenylmethylsiloxane, 30 m length, 0.25 mm inner diameter, and 0.25 µm film thickness, Agilent technologies). The injector temperature was 270 °C and the oven temperature was programmed from 60 (0 min) to 200 °C at a rate of 5 °C /min. Helium was selected as the carrier gas, while the flow rate was set to 1 mL/min with an injection volume of 1 µL. However, the mass spectrometer was set to ionization mode with a voltage of 70 eV. The interfacial temperature was adjusted to 280 °C, and the mass range was between 35 and 500 m/z. The oleoresin components were identified based on a comparison of their retention indices (C_7_ to C_20_ n-alkanes) and their mass spectral fragmentation patterns, which were used to calculate the Kovats Indices from the gas chromatographic analysis. The Kovats indices were calculated using the Kovates equations^44,45^.

### FTIR spectroscopy

FTIR spectroscopy of oleoresin was performed using a Perkin-Elmer FTIR spectrometer (Spectrum GX, USA). The measurement was performed according to the previous work with some modifications^46,47^. Briefly, the samples were on the KBr disc to scan the spectral range from 400 to 4000 cm^−1^ and 50 scans were measured with a resolution of 1 cm^−1^.

### Statistical analysis

Data were expressed as mean ± standard deviation. Experimental design and regression analysis were done with Design-Expert software version 13 (Statease Inc., Minneapolis, MN, USA), which was used for response surface method. Statistical analysis was conducted using Origin 8.0 and statistical significance was set at *p* < 0.05. The fitness of the proposed model was calculated by evaluating the coefficient of determination (R^2^), lack of fit, and F-value based on analysis of variance (ANOVA).

### Research involving plants

The authors declare that the study on plants in this research, including the collection of plant materials, complies with relevant institutional, national, and international guidelines and legislation.

### Ethics approval and consent to participate

The authors will follow the Ethical Responsibilities of Authors and COPE rules. On behalf of all co-authors, I believe the participants are giving informed consent to participate in this study.

## Results and discussion

### Selection of DES solvents

As mentioned earlier, DES solvents have been widely used to extract antioxidants, polyphenols, and many polar compounds from plant materials. In the present work, 6 types of DES were used for the extraction of *F. gummosa* oleoresin (Table 2). The results showed that the oleoresin yield was highest when the solvents CL/formic acid and CL/formamide were used. This might be due to the stronger hydrogen bonding of DES solvents developed by choline chloride and formic acid or formamide, which can form a strong intermolecular bond with oleoresin in *Ferula gummosa*^48^*.* However, the yield increased when the molar ratio of the solvents CL/formic acid and CL/formamide was changed from 2:1 to 3:1. These results may be attributed to acid hydrolysis of the bonds between cell wall components due to the acidifying properties of formic acid in DES (i.e., a higher amount of formic acid was present in DES), which released the oleoresin into the extraction medium and increased the extraction yield, consistent with previous results^49^. Increasing the molar ratio of choline chloride (data not shown) was also decreased the extraction rate of oleoresin, which is due to the fact that the increasing ratio of CL increases the pH of the solvent and thus affects the oleoresin yield. Therefore, the highest extraction amount of oleoresin was achieved with the ratios shown in Table 2. Consequently, the solvent DES, consisting of CL/formic acid and CL/formamide, was selected for further research.

**Table 2:** *Ferula gummosa* oleoresin yields corresponding to different DES solvents.

HBA: HBD	Molar ratio	Oleoresin yield (%)
CL: Urea	2:1	13.62 ± 0.94
CL: Lactic acid	2:1	13.28 ± 0.78
CL: Formic acid	3:1	15.37 ± 1.16
CL: Acetic acid	2:1	13.83 ± 1.14
CL: Glycerol	2:1	11.61 ± 0.61
CL: Formamide	3:1	15.68 ± 1.25

### Single factor experiments

**Figure 1:**
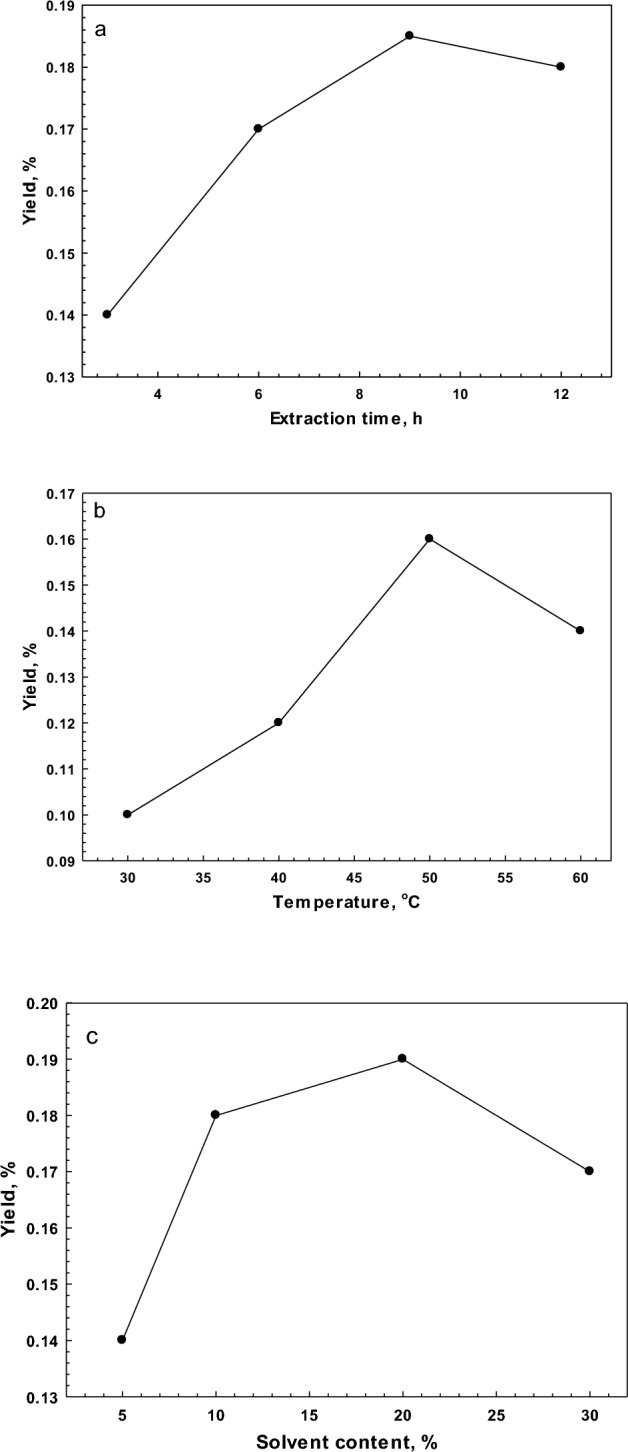
Effect of single factor on the yield extraction of oleoresin. (**a**) Extraction time (h). (**b**) Extraction temperature (°C), and (**c**) solvent content (%).

The effects of time, temperature, and the ratio of DES solvent to *F. gummosa* on the extraction yield of *F. gummosa* are shown in Fig. 1. As can be seen, the extraction yield increased with time and temperature; however, higher temperatures and longer periods were not used due to the changes in the physical properties of DES. The results showed that the oleoresin yield increased significantly (*P* < 0.05) when the extraction time was increased from 3.0 to 9.0 h, and the maximum yield (18.67%) was obtained at 9 h (Fig. 1a). This is due to the time (3 h) required to completely release the oleoresin into the extraction medium. During the process, the solution of the DES penetrated the *F. gummosa* matrices, hydrolyzed the bonds between the solubilized cell walls, and then diffused out of the cell wall. However, when the extraction time was increased up to 10 h, no further increase in yield was observed. When the temperature was increased from 30 to 50 °C, the yield increased significantly (*P* < 0.05) from 9.2 to 17.4% (Fig. 1b). This increasing trend in yield with increasing temperature was related to the increase in flowability of oleoresin. The growth in temperature decreased the viscosity reduction the diffusion of DES, which assisted to destroy the chemical bond between oleoresin and cell walls, thus improving the dissolution of oleoresin. During extraction, heat enhanced the rupture of bonds between cell walls. The result is similar to previous findings in the extraction of polysaccharides from black mulberry, where the yield increased when the extraction temperature was raised from 60 to 90 °C^50^. As shown in Fig. 1c, the extraction yield decreased when the ratio of DES solvent to *F. gummosa* exceeded 20%, while at 10%, the oleoresin yield reached a maximum of 18%. Based on the results of the one single-factor experiment, the ranges of response surface methodology (RSM) factors were applied for further evaluation as follows: DES ratio of solvent to *F. gummosa* (5–20%), extraction temperature (30–50 °C), and extraction time (3–9 h).

### Response surface optimization

The response surface method was generally applied in optimization of the extraction parameters. Thus, the optimal conditions for extraction of oleoresin were evaluated using RSM. The experimental matrix design with a total of 34 runs (17 runs for formic acid and formamide) between the experimental and predicted values. The results showed good agreement between experimental and predicted values. As shown in Table 3, multiple regression models were fitted to the data using Design-Expert software and the quadratic polynomial regression equation corresponding to the coding independent variable of extraction yield (Y) of the samples was obtained: Y = 18.01 + 0.52A + 1.57B + 0.76AB − 0.49AC − 0.75BC − 0.44A^2^ − 2.5B^2^ − 2.38C^2^.

**Table 3:** BBD with actual and predicted values for the yield of the *F. gummosa* Oleoresin.

Run	A: Time (h)	B: Temperature (°C)	C: Ratio (%)	D: DES solvent	Yield (%)	
					Predict value	Actual value
1	9	40	20	Formamide	17.24	17.1
2	6	40	12.5	Formic	15.23	15.64
3	3	40	5	Formic	13.74	14.04
4	6	30	5	Formic	14.12	14.52
5	6	30	20	Formic	17.05	16.84
6	3	40	20	Formic	15.46	15.53
7	6	50	5	Formamide	14.87	14.28
8	6	50	5	Formic	16.12	16.78
9	3	40	5	Formamide	15.78	15.06
10	9	40	20	Formic	19.45	18.65
11	9	40	5	Formamide	16.23	16.35
12	6	30	20	Formamide	15.75	15.19
13	3	30	12.5	Formic	15.27	14.35
14	3	30	12.5	Formamide	14.12	13.54
15	6	40	12.5	Formic	16.28	15.62
16	6	40	12.5	Formamide	17.06	16.67
17	9	40	5	Formic	18.06	17.82
18	6	40	12.5	Formamide	16.34	16.54
19	6	50	20	Formamide	18.47	18.47
20	6	50	20	Formic	18.11	18.65
21	3	50	12.5	Formamide	15.76	15.06
22	6	40	12.5	Formamide	16.59	16.49
23	9	30	12.5	Formamide	16.28	16.1
24	6	40	12.5	Formic	17.96	17.82
25	9	30	12.5	Formic	17.16	17.54
26	9	50	12.5	Formamide	18.65	18.27
27	3	40	20	Formamide	16.59	16.43
28	6	30	5	Formamide	17.93	17.85
29	3	50	12.5	Formic	16.45	16.78
30	6	40	12.5	Formic	17.89	17.24
31	6	40	12.5	Formic	17.34	17.68
32	9	50	12.5	Formic	18.46	18.54
33	6	40	12.5	Formamide	16.37	16.95
34	6	40	12.5	Formamide	16.25	16.84

The analysis of variance of the quadratic response surface model is presented in Table 4. The mathematical model *P* < 0.05 revealed the significance of the model. The primary terms B, B^2^, and C^2^ had a significant effect on the quadratic terms; the misfit had no significant effect (*P* < 0.05). Model R^2^ = 0.8542, R^2^Adj = 0.6562, indicating that 85.42% of the change in response value came from the selected variables. This equation fitted well with the actual situation, and could be used to analyze the test results instead of the real test^51^. The coefficient of variation (CV) represents the ratio of the standard deviation to the mean, which shows the extent of variability in data. The model’s coefficient of variation was 5.67%, indicating that the experiment had good accuracy and high reliability. According to the F value, the order of influence of each factor on the extraction rate of oleoresin was: extraction temperature > solvent ratio > extraction time.

**Table 4:** Response surface quadratic model variance analysis.

Source	Sum of squares	d_f_	Mean square	F-value	*p* Value	
*Model*	48.45	13	3.73	4.12	0.0023	Significant
A-time	23.96	1	23.96	26.52	< 0.0001	
B-Temperature	7.43	1	7.43	8.22	0.0095	
C-Ratio	6.45	1	6.45	7.14	0.0146	
D-Solvent ratio	1.38	1	1.38	1.53	0.2308	
AB	0.0760	1	0.0760	0.0842	0.7747	
AC	0.2048	1	0.2048	0.2266	0.6392	
AD	1.06	1	1.06	1.17	0.2915	
BC	5.12	1	5.12	5.67	0.0274	
BD	1.05	1	1.05	1.16	0.2937	
CD	0.5112	1	0.5112	0.5658	0.4607	
A^2^	0.9635	1	0.9635	1.07	0.3141	
B^2^	0.1610	1	0.1610	0.1781	0.6775	
C^2^	0.0123	1	0.0123	0.0136	0.9082	
*Residual*	18.07	20	0.9036			
Lack of fit	13.17	12	1.10	1.79	0.2072	Not significant
Pure error	4.90	8	0.6124			
*Cor total*	66.52	33				

**Figure 2:**
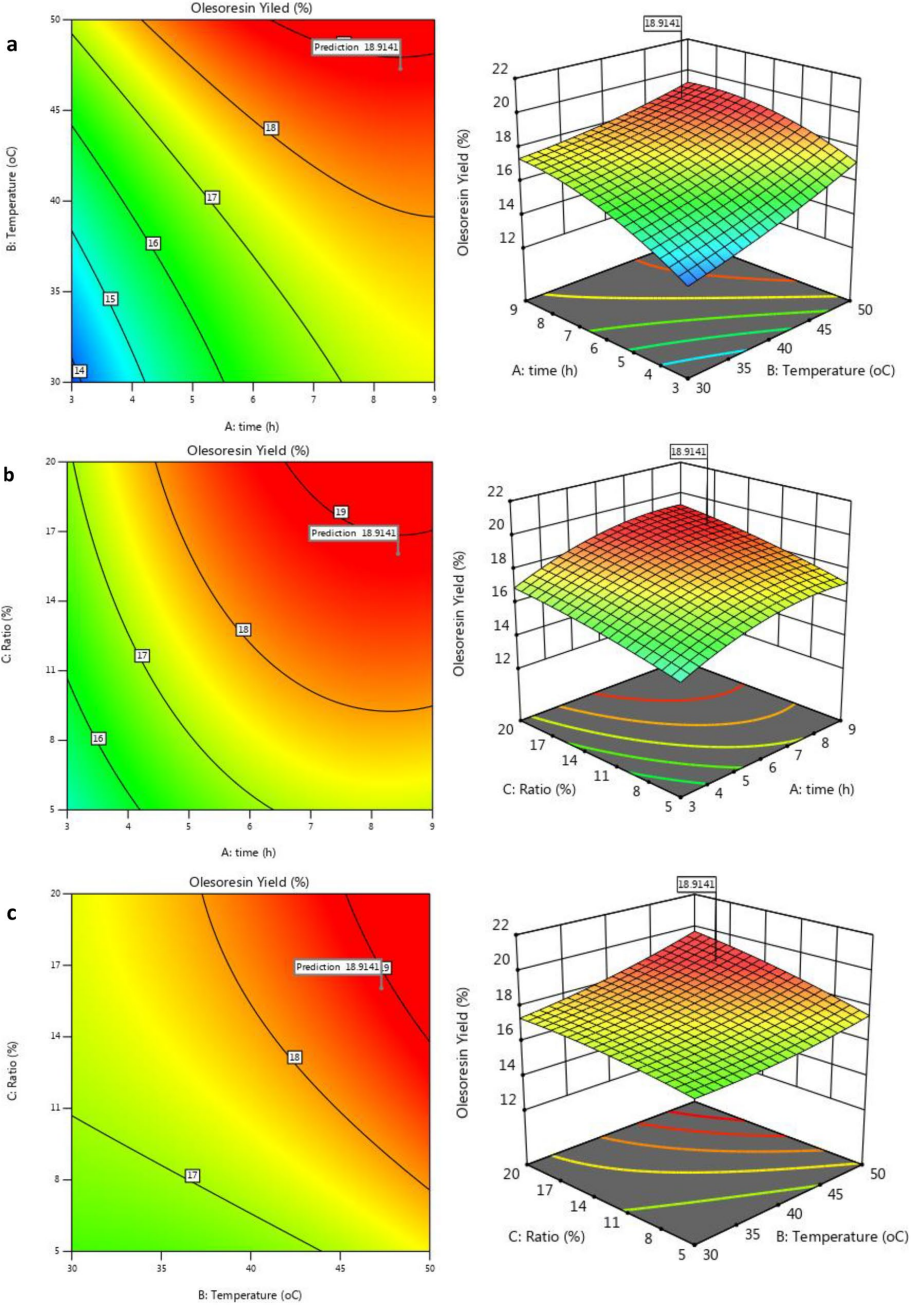
2D response contour map (left hand) and 3D surface (right hand) of DES assisted extraction. (**a**) Time–temperature, (**b**) Time-ratio and (**c**) Temperature-ratio interactions.

The interaction between two independent variables could be directly observed from the 3D response surface map and the 2D response contour map. When the contour line was saddle-shaped or oval, the interaction between two factors was significant, whereas when the contour line was round, the interaction was not significant. The higher the slope of response surface, the more significant the interaction was. The effects of extraction time (A), extraction temperature (B), and solvent content (C) on the extraction rate of oleoresin from *F. gummosa* are shown in Fig. 2. The slope of the response surface of B was the highest, indicating that B had the greatest effect on the extraction rate. The contour lines of AB and AC were elliptical, indicating significant interactions between extraction time and extraction temperature as well as reagent content. The contour line of BC was nearly a circular circle, indicating little interaction between extraction temperature and reagent content. All these results were in agreement with the results of ANOVA in Table 4.

**Figure 3:**
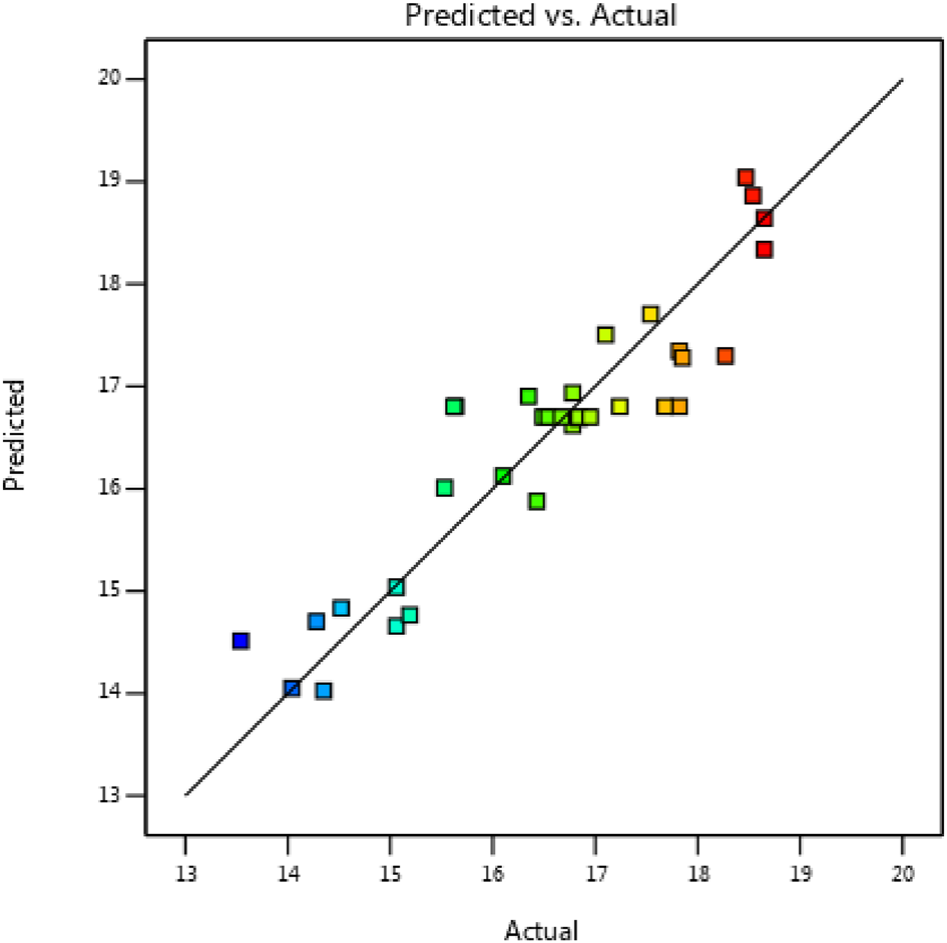
Actual value and predicted value fitting model diagram.

When the fitting model was checked (Fig. 3), the predicted value and the simulated value were almost on a straight line, indicating that the model was in good agreement with the actual situation. Considering the extraction efficiency, energy saving effect and feasibility of the experiment, the optimal extraction parameters were proposed as follows: extraction time 6 h, temperature 40 °C, solvent content 12.5% (w/v), and the predicted yield was 17.24%. Independent t-test analysis showed that there was no significant difference between the experimental value (17.24%) and the predicted value (*p* > 0.05). Therefore, the model could be used to optimize the extraction process of oleoresin from *F. gummosa* in a certain range, and the optimal extraction parameters were determined.

### Chemical composition of *F. gummosa* oleoresin

**Figure 4:**
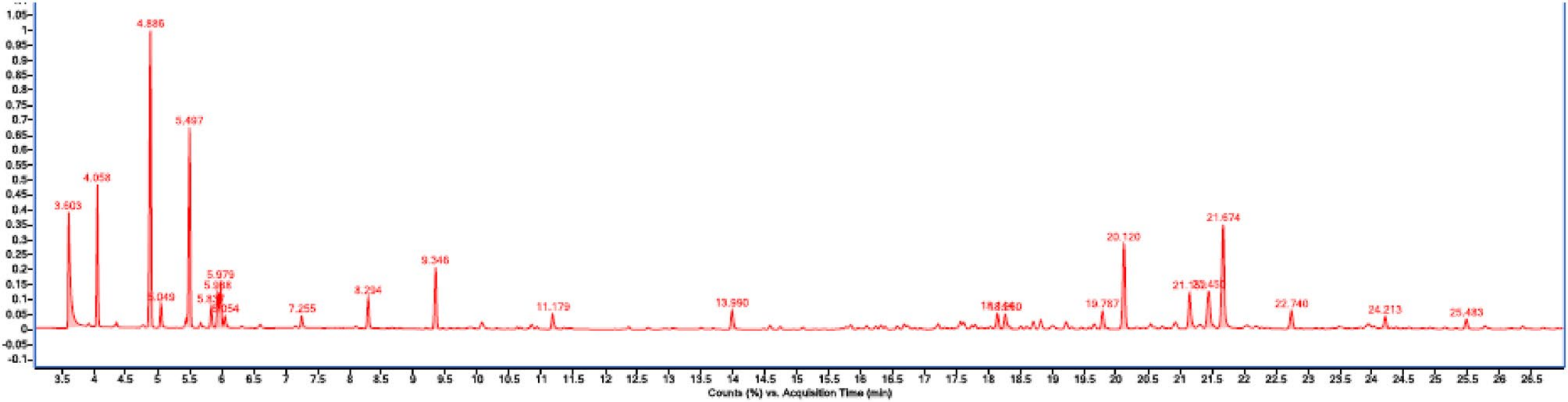
GC analysis of *F. gummosa* oleoresin.

The results of the GC–MS analysis of the *F. gummosa* oleoresin are shown in Table 5. The compounds are ranked in order of retention time. A typical of GC analysis of *F. gummosa* oleoresin is also shown in Fig. 4. A total of 20 compounds were identified in the oleoresin, with β-pinene (40.27%), cylcofenchenes (11.93%), and α-pinene (7.53%) being the major components. The main group was monoterpene hydrocarbons (62.70%) including α-thujene, α-pinene, and β-pinene. The other main components of oleoresin were β-cymene (8.2%) and α-thujene (10.49%). The results showed that the monoterpene fraction accounted for 60% of the oil components and sequiterpenes were the remaining components (~ 40%), which is consistent with a previous preliminary study^6^. The oil contained low levels of limonene (2.11%), β-phellandrene (2.97), terpinene (1%), m-cymene (1.39%) and β-ocimene (1%)^52^. Similar results were reported that α-pinene (27.27%), β-pinene (43.78%), and β-myrcene (3.37%) were the three major components of *F. gummosa*. In another study, α-pinene, β-pinene, linalool, α-terpinolene, delta-3-carene, and terpinolene were the most important components of *F.gummosa* oleoresin^7,53^. These differnces in results may be due to factors such as geographical origin, harvesting time, and extraction method of oleoresin^2,6,9,10^.

**Table 5:** Chemical composition of *F. Gummosa* oleoresin by GC–MS analysis.

Compound	Retention time (min)	Composition (%)
α-Thujene	3.603	10.49
α-Pinene	4.058	7.53
β-Pinene	4.886	40.27
β-Cymene	5.049	8.21
Cylcofenchene	5.497	11.93
m-Cymene	5.837	1.39
D-limonene	5.938	2.11
β-Phellandrene	5.979	2.97
Trans-β-Ocimene	6.054	0.92
Terpinene	7.255	0.9
α-Cyclogeraniol	8.294	2.22
α-Terpinyl acetate	13.99	1.65
Cadinene	18.144	1.49
Cadina-3.9-diene	18.261	1.69
Junenol	19.787	1.68
Guaiol	20.127	1.44
Cadinol	21.152	2.29
Eudesmol	21.450	4.33
Bulnesol	21.674	2.61
Guaiac acetate	22.740	0.69

### *Ferula gummosa* oleoresin characterization by FTIR

**Figure 5:**
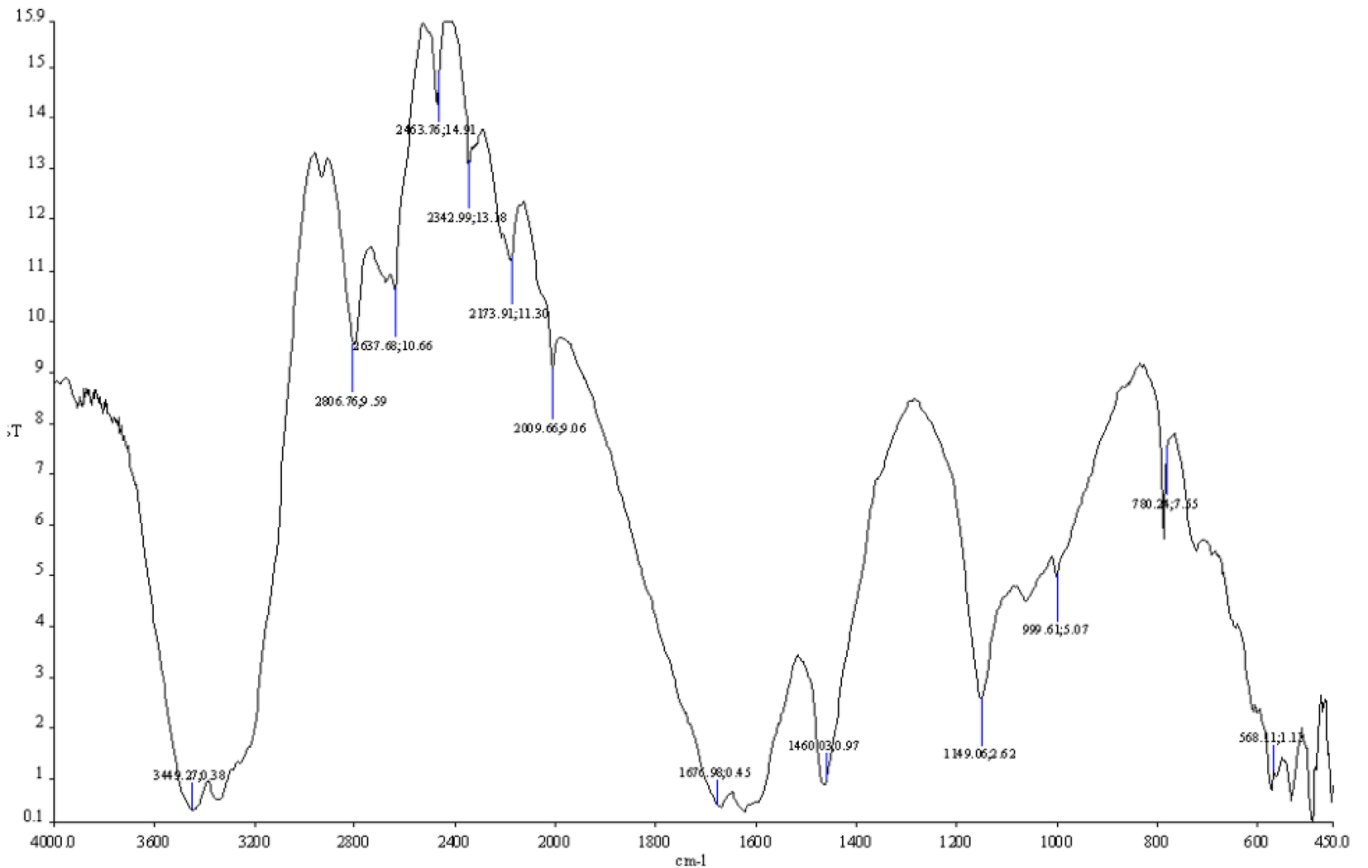
Structural characterization of *F. gummosa* oleoresin through infrared spectroscopy.

The infrared spectrum of the oleoresin obtained under optimal extraction conditions is illustrated in Fig. 5. FTIR was performed to determine structural information about the functional groups and the orientation of the groups in the oleoresin of *F. gummosa*. A broad band in the region of 3400 cm^−1^ indicates stretching vibration of the hydroxyl group (O–H). A similar result was reported for the FTIR spectrum of pomegranate peel pectin^54^. The absorption peak of the O–H stretching vibrations was found at 3449 cm^−1^. The small band was found in the 2806 cm^−1^ region represents the C–H absorption, which includes CH, CH_2_ and CH_3_ stretching and bending vibrations. It was reported that the peaks at 2800 cm^−1^ and 1320 cm^−1^ are associated with the CH stretching vibrations of CH_2_ groups^55^. Moreover, a small band at around 2637 cm^−1^ for *F. gummosa* oleoresin indicates the existence of an aliphatic C–H bond. This C–H absorption and aliphatic C–H bond could be due to the methyl ester groups (OCH_3_). In addition, two peaks were indicated between 1678 and 1460 cm^−1^, characteristics of an ester carbonyl group (C=O) and an unesterified carboxylate ion (COO–), respectively^56^. The prominent bands were also detected in the range between 1300 and 1000 cm^−1^ attributed to C–O stretching in C–O–C and C–O–H. These could be expected owing to the typical presence of pyranose ring in oleoresin, which linked with one another via C–O–C glycosidic linkages and side group C–O–H linked bonds. As mentioned by Yang et al. (2018), the significant absorption band located at 1677 cm^−1^ was assigned to C–O stretching vibration of ester carbonyl, and the absorption band at around 1677 cm^−1^ was represented by C–O stretching vibration of non-methylated carboxyl in pomegranate peel pectin^54^. In addition, strong absorption peaks between 1149 and 1018 cm^−1^ indicated the potential presence of pyranose ring. The absorption bands in the range of 1300 cm^−1^ and 1450 cm^−1^ for both high and low methyl groups were assigned to CH_3_ corresponding to asymmetric stretching vibrations, while the bands around 1020 cm^−1^ were allocated to the saccharide structure (C–O–C). IR spectrum for DES (Fig. 5) was also determined in order to check any DES residues in the extracted oleoresin. The result showed that the IR spectra of DES were not found in the IR spectra of oleoresin. Therefore, it was assumed that no DES residue was detectable in the oleoresin.

## Conclusion

**Figure 6:**
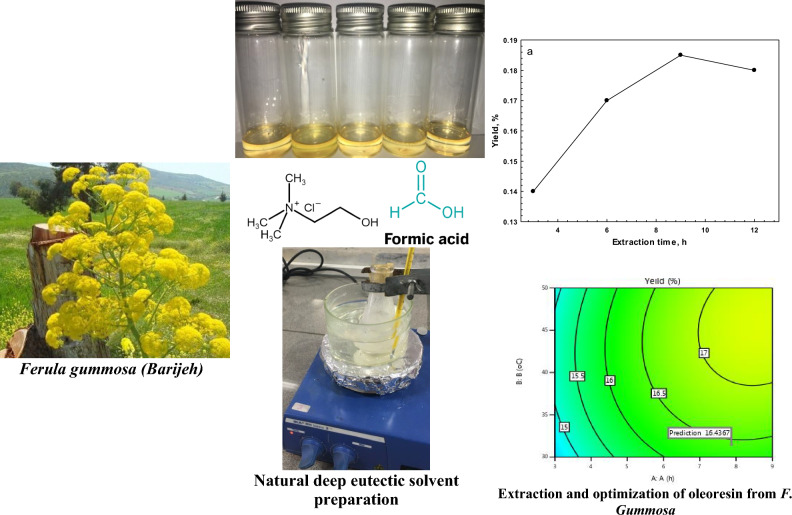
Summary of the research work on natural deep eutectic solvent (NADES) in oleoresin extraction from *Ferula gummosa* roots.

Summary of the work was provided in a separate figure (Fig. 6). The use of DES proved to be an effective extraction medium for the extraction of oleoresin from *F. gummosa*. Optimal conditions were determined: to be a solvent ratio 12.5% (w/v), an extraction temperature of 40 °C, extraction time of 6 h, and a molar ratio of the components of DES 3:1. Among the different DES solvents, choline chloride/formic acid and choline chloride/formamide in the ratio of 3:1 showed the highest yield of in the extraction of oleoresin. The optimum condition gave a yield of oleoresin of 17.24%. The GC–MS analysis also exhibited its potential as a functional oleoresin that could be used as a food ingredient. The oleoresin was mainly composed of β-pinene (40.27%), cylcofenchen (11.93%) and α-pinene (7.53%), with the main group being monoterpene hydrocarbons (62.70%), including α-thujene, α-pinene and β-pinene potential. These findings demonstrated that oleoresin extracted from *F. gummosa* could be explored as novel promising natural pharmaceutical ingredient that can be extracted with eutectic solvents.

## Data Availability

All data are presented in the manuscript.

## References

[1] Sadraei H, Asghari GR, Hajhashemi V, Kolagar A, Ebrahimi M (2001). Spasmolytic activity of essential oil and various extracts of *Ferula gummosa* Boiss. on ileum contractions. Phytomedicine.

[2] Abedi D, Jalali M, Sadeghi N (2009). Composition and antimicrobial activity of oleogumresin of *Ferula gumosa* Bioss. essential oil using Alamar BlueTM. Res. Pharm. Sci..

[3] Mortazaienezhad F, Sadeghian MM (2006). Investigation of compounds from galbanum (*Ferula gummosa*) boiss. Asian J. Plant Sci..

[4] Javidnia K, Miri R, Kamalinejad M, Edraki N (2005). Chemical composition of *Ferula persica* Wild. essential oil from Iran. Flavour Fragr. J..

[5] Mohammadzadeh MJ, Emam JZ, Safari M, Mousavi M, Ghanbarzadeh B, Philips GO (2007). Physicochemical and emulsifying properties of Barijeh (*Ferula gumosa*) gum. Iran. J. Chem. Chem. Eng. (IJCCE).

[6] Jalali HT, Ebrahimian ZJ, Evtuguin DV, Neto CP (2011). Chemical composition of oleo-gum-resin from *Ferula gummosa*. Ind. Crops Prod..

[7] Ghasemi Y, Faridi P, Mehregan I, Mohagheghzadeh A (2005). *Ferula gummosa* fruits: An aromatic antimicrobial agent. Chem. Nat. Compd..

[8] Iranshahy M, Iranshahi M (2011). Traditional uses, phytochemistry and pharmacology of asafoetida (Ferula assa-foetida oleo-gum-resin)—A review. J. Ethnopharmacol..

[9] Jalali HT, Petronilho S, Villaverde JJ, Coimbra MA, Domingues MRM, Ebrahimian ZJ (2012). Deeper insight into the monoterpenic composition of *Ferula gummosa* oleo-gum-resin from Iran. Ind. Crops Prod..

[10] Jalali HT, Petronilho S, Villaverde JJ, Coimbra MA, Domingues MRM, Ebrahimian ZJ (2013). Assessment of the sesquiterpenic profile of *Ferula gummosa* oleo-gum-resin (galbanum) from Iran. Contributes to its valuation as a potential source of sesquiterpenic compounds. Ind. Crops Prod..

[11] Chemat F, Vian MA (2014). Alternative Solvents for Natural Products Extraction.

[12] Ahmad I, Pertiwi AS, Kembaren YH, Rahman A, Munâ A (2018). Application of natural deep eutectic solvent-based ultrasonic assisted extraction of total polyphenolic and caffeine content from Coffe Beans (*Coffea Beans* L.) for instant food products. J. Appl. Pharm. Sci..

[13] Vian M, Breil C, Vernes L, Chaabani E, Chemat F (2017). Green solvents for sample preparation in analytical chemistry. Curr. Opin. Green Sustain. Chem..

[14] Espino M, de los Ángeles Fernández M, Gomez FJV, Silva MF (2016). Natural designer solvents for greening analytical chemistry. TrAC Trends Anal. Chem..

[15] Pan M, Zhao G, Ding C, Wu B, Lian Z, Lian H (2017). Physicochemical transformation of rice straw after pretreatment with a deep eutectic solvent of choline chloride/urea. Carbohydr. Polym..

[16] de los Ángeles Fernández M, Espino M, Gomez FJV, Silva MF (2018). Novel approaches mediated by tailor-made green solvents for the extraction of phenolic compounds from agro-food industrial by-products. Food Chem..

[17] Paiva A, Craveiro R, Aroso I, Martins M, Reis RL, Duarte ARC (2014). Natural deep eutectic solvents–solvents for the 21st century. ACS Sustain Chem. Eng..

[18] Dai Y, Witkamp G-J, Verpoorte R, Choi YH (2015). Tailoring properties of natural deep eutectic solvents with water to facilitate their applications. Food Chem..

[19] Shahbaz K, Baroutian S, Mjalli FS, Hashim MA, AlNashef IM (2012). Densities of ammonium and phosphonium based deep eutectic solvents: Prediction using artificial intelligence and group contribution techniques. Thermochim. Acta..

[20] Maugeri Z, de María PD (2012). Novel choline-chloride-based deep-eutectic-solvents with renewable hydrogen bond donors: Levulinic acid and sugar-based polyols. RSC Adv..

[21] Ribeiro BD, Coelho MAZ, Marrucho IM (2013). Extraction of saponins from sisal (*Agave sisalana*) and juá (*Ziziphus joazeiro*) with cholinium-based ionic liquids and deep eutectic solvents. Eur. Food Res. Technol..

[22] Syakfanaya AM, Saputri FC, Mun’im A (2019). Simultaneously extraction of caffeine and chlorogenic acid from *Coffea canephora* bean using natural deep eutectic solvent-based ultrasonic assisted extraction. Pharmacogn. J..

[23] Yuniarti E, Saputri FC, Munâ A (2019). Application of the natural deep eutectic solvent choline chloride-sorbitol to extract chlorogenic acid and caffeine from green coffee beans (*Coffea canephora*). J. Appl. Pharm. Sci..

[24] Nadia J, Shahbaz K, Ismail M, Farid MM (2018). Approach for polygodial extraction from *Pseudowintera colorata* (Horopito) leaves using deep eutectic solvents. ACS Sustain. Chem. Eng..

[25] Cao J, Chen L, Li M, Cao F, Zhao L, Su E (2018). Efficient extraction of proanthocyanidin from Ginkgo biloba leaves employing rationally designed deep eutectic solvent-water mixture and evaluation of the antioxidant activity. J. Pharm. Biomed. Anal..

[26] Machmudah S, Lestari SD, Kanda H, Winardi S, Goto M (2018). Subcritical water extraction enhancement by adding deep eutectic solvent for extracting xanthone from mangosteen pericarps. J Supercrit. Fluids.

[27] Křížek T, Bursová M, Horsley R, Kuchař M, Tůma P, Čabala R (2018). Menthol-based hydrophobic deep eutectic solvents: Towards greener and efficient extraction of phytocannabinoids. J. Clean. Prod..

[28] Athanasiadis V, Grigorakis S, Lalas S, Makris DP (2018). Highly efficient extraction of antioxidant polyphenols from *Olea europaea* leaves using an eco-friendly glycerol/glycine deep eutectic solvent. Waste Biomass Valoriz..

[29] Liew SQ, Ngoh GC, Yusoff R, Teoh WH (2018). Acid and deep eutectic solvent (DES) extraction of pectin from pomelo (*Citrus grandis* (L.) Osbeck) peels. Biocatal. Agric. Biotechnol..

[30] Ma W, Row KH (2017). Optimized extraction of bioactive compounds from *Herba Artemisiae Scopariae* with ionic liquids and deep eutectic solvents. J. Liq. Chromatogr. Relat. Technol..

[31] De Faria ELP, Do Carmo RS, Cláudio AFM, Freire CSR, Freire MG, Silvestre AJD (2017). Deep eutectic solvents as efficient media for the extraction and recovery of cynaropicrin from *Cynara cardunculus* L. leaves. Int. J. Mol. Sci..

[32] Dai Y, Verpoorte R, Choi YH (2014). Natural deep eutectic solvents providing enhanced stability of natural colorants from safflower (*Carthamus tinctorius*). Food Chem..

[33] Huang Y, Feng F, Jiang J, Qiao Y, Wu T, Voglmeir J (2017). Green and efficient extraction of rutin from tartary buckwheat hull by using natural deep eutectic solvents. Food Chem..

[34] Wang T, Jiao J, Gai Q-Y, Wang P, Guo N, Niu L-L (2017). Enhanced and green extraction polyphenols and furanocoumarins from Fig (*Ficus carica* L.) leaves using deep eutectic solvents. J. Pharm. Biomed. Anal..

[35] Zhao B-Y, Xu P, Yang F-X, Wu H, Zong M-H, Lou W-Y (2015). Biocompatible deep eutectic solvents based on choline chloride: Characterization and application to the extraction of rutin from *Sophora japonica*. ACS Sustain Chem. Eng..

[36] Wei Z, Qi X, Li T, Luo M, Wang W, Zu Y (2015). Application of natural deep eutectic solvents for extraction and determination of phenolics in *Cajanus cajan* leaves by ultra performance liquid chromatography. Sep. Purif. Technol..

[37] Zahrina I, Nasikin M, Krisanti E, Mulia K (2018). Deacidification of palm oil using betaine monohydrate-based natural deep eutectic solvents. Food Chem..

[38] Manic MS, Najdanovic-Visak V, da Ponte MN, Visak ZP (2011). Extraction of free fatty acids from soybean oil using ionic liquids or poly (ethyleneglycol) s. Aiche J..

[39] Depoorter J, Mourlevat A, Sudre G, Morfin I, Prasad K, Serghei A (2019). Fully biosourced materials from combination of choline chloride-based deep eutectic solvents and guar gum. ACS Sustain. Chem. Eng..

[40] Wei Z-F, Wang X-Q, Peng X, Wang W, Zhao C-J, Zu Y-G (2015). Fast and green extraction and separation of main bioactive flavonoids from *Radix Scutellariae*. Ind. Crops Prod..

[41] Peng X, Duan M-H, Yao X-H, Zhang Y-H, Zhao C-J, Zu Y-G (2016). Green extraction of five target phenolic acids from *Lonicerae japonicae* Flos with deep eutectic solvent. Sep. Purif. Technol..

[42] Wang M, Wang J, Zhou Y, Zhang M, Xia Q, Bi W (2017). Ecofriendly mechanochemical extraction of bioactive compounds from plants with deep eutectic solvents. ACS Sustain. Chem. Eng..

[43] Abbott AP, Boothby D, Capper G, Davies DL, Rasheed RK (2004). Deep eutectic solvents formed between choline chloride and carboxylic acids: Versatile alternatives to ionic liquids. J. Am. Chem. Soc..

[44] Sandra, P. & Bicchi, C. Capillary gas chromatography in essential oil analysis (1987).

[45] Fernández AG, Adams MR, Fernández-Díez MJ (1997). Table olives: Production and processing.

[46] Rafe A, Razavi SMA (2015). Effect of thermal treatment on chemical structure of β-lactoglobulin and basil seed gum mixture at different states by ATR-FTIR spectroscopy. Int. J. Food. Prop..

[47] Shahbazi M, Rajabzadeh G, Rafe A, Ettelaie R, Ahmadi SJ (2016). The physico-mechanical and structural characteristics of blend film of poly (vinyl alcohol) with biodegradable polymers as affected by disorder-to-order conformational transition. Food Hydrocoll..

[48] Chen W, Xue Z, Wang J, Jiang J, Zhao X, Mu T (2018). Investigation on the thermal stability of deep eutectic solvents. Acta Phys. Chim. Sin..

[49] Shafie MH, Yusof R, Gan C-Y (2019). Deep eutectic solvents (DES) mediated extraction of pectin from *Averrhoa bilimbi*: Optimization and characterization studies. Carbohydr. Polym..

[50] Wang W, Li X, Bao X, Gao L, Tao Y (2018). Extraction of polysaccharides from black mulberry fruit and their effect on enhancing antioxidant activity. Int. J. Biol. Macromol..

[51] Shang X, Chu D, Zhang J, Zheng Y, Li Y (2021). Microwave-assisted extraction, partial purification and biological activity in vitro of polysaccharides from bladder-wrack (*Fucus vesiculosus*) by using deep eutectic solvents. Sep. Purif. Technol..

[52] Rios J-L, Recio MC (2005). Medicinal plants and antimicrobial activity. J. Ethnopharmacol..

[53] Eftekhar F, Yousefzadi M, Borhani K (2004). Antibacterial activity of the essential oil from *Ferula gummosa* seed. Fitoterapia.

[54] Yang X, Nisar T, Hou Y, Gou X, Sun L, Guo Y (2018). Pomegranate peel pectin can be used as an effective emulsifier. Food Hydrocoll..

[55] He L, Zhang X, Xu H, Xu C, Yuan F, Knez Ž (2012). Subcritical water extraction of phenolic compounds from pomegranate (*Punica granatum* L.) seed residues and investigation into their antioxidant activities with HPLC–ABTS+ assay. Food Bioprod. Process..

[56] Manrique GD, Lajolo FM (2002). FT-IR spectroscopy as a tool for measuring degree of methyl esterification in pectins isolated from ripening papaya fruit. Postharvest Biol. Technol..

